# Glycogen Synthase Kinase-3 Beta (GSK3β) as a Potential Drug Target in Regulating Osteoclastogenesis: An Updated Review on Current Evidence

**DOI:** 10.3390/biom14040502

**Published:** 2024-04-21

**Authors:** Sok Kuan Wong

**Affiliations:** Department of Pharmacology, Faculty of Medicine, Universiti Kebangsaan Malaysia, Jalan Yaacob Latif, Bandar Tun Razak, Cheras, Kuala Lumpur 56000, Malaysia; sokkuan@ukm.edu.my; Tel.: +60-3-9145-9566

**Keywords:** GSK3β, bone loss, bone resorption, osteoclast, osteoporosis

## Abstract

Glycogen synthase kinase 3-beta (GSK3β) is a highly conserved protein kinase originally involved in glucose metabolism, insulin activity, and energy homeostasis. Recent scientific evidence demonstrated the significant role of GSK3β in regulating bone remodelling through involvement in multiple signalling networks. Specifically, the inhibition of GSK3β enhances the conversion of osteoclast progenitors into mature osteoclasts. GSK3β is recognised as a pivotal regulator for the receptor activator of nuclear factor-kappa B (RANK)/receptor activator of nuclear factor-kappa B ligand (RANKL)/osteoprotegerin (OPG), phosphatidylinositol-3-kinase (PI3K)/protein kinase B (AKT), nuclear factor-kappa B (NF-κB), nuclear factor-erythroid 2-related factor 2 (NRF2)/Kelch-like ECH-associated protein 1 (KEAP1), canonical Wnt/beta (β)-catenin, and protein kinase C (PKC) signalling pathways during osteoclastogenesis. Conversely, the inhibition of GSK3β has been shown to prevent bone loss in animal models with complex physiology, suggesting that the role of GSK3β may be more significant in bone formation than bone resorption. Divergent findings have been reported regarding the efficacy of GSK3β inhibitors as bone-protecting agents. Some studies demonstrated that GSK3β inhibitors reduced osteoclast formation, while one study indicated an increase in osteoclast formation in RANKL-stimulated bone marrow macrophages (BMMs). Given the discrepancies observed in the accumulated evidence, further research is warranted, particularly regarding the use of GSK3β silencing or overexpression models. Such efforts will provide valuable insights into the direct impact of GSK3β on osteoclastogenesis and bone resorption.

## 1. Introduction

Bone is a highly dynamic connective tissue which undergoes balanced bone remodelling under physiology conditions. Osteoblasts and osteoclasts are the two main cells that specialise in new bone formation and aged bone resorption, respectively [1]. Osteoporosis is a chronic and progressive bone disease characterised by the loss of bone mineral density, leading to weakened and brittle bones and thereby increasing fracture risk. This condition arises from osteoclastic bone resorption, surpassing osteoblastic bone formation [2]. Osteoclastogenesis is a multi-step process involving osteoclast progenitor commitment proliferation, differentiation, and fusion to form multinucleated mature osteoclasts. Osteoblasts mediate osteoclastogenesis mainly through the secretion of two key cytokines, macrophage colony-stimulating factor (M-CSF) and receptor activator of nuclear factor-kappa B ligand (RANKL). The binding of M-CSF to the colony-stimulating factor-1 receptor (CSF-1R or c-Fms) induces the receptor activator of nuclear factor-kappa B (RANK) expression and its surface localisation. Subsequently, RANKL binds to RANK, triggering the recruitment of tumour necrosis factor receptor-associated factor 6 (TRAF6) and activation of downstream signalling cascades to initiate monocyte-to-osteoclast differentiation, monocyte multinucleation, as well as activation and survival of mature osteoclasts. The nuclear factor of activated T cells cytoplasmic 1 (NFATc1) is dephosphorylated, and this is followed by its translocation into the nucleus for transcription of osteoclast-specific genes, including tartrate-resistant acid phosphatase (TRAP), cathepsin K (CTSK), osteoclast-associated receptor (OSCAR), and vacuolar-type proton ATPase subunit d2 (Atp6v0d2). Osteoprotegerin (OPG) is a soluble decoy receptor for RANKL, preventing RANKL/RANK interaction and eventually monocyte-to-osteoclast differentiation and osteoclast maturation [3].

Glycogen synthase kinase-3 beta (GSK3β) is a ubiquitous serine/threonine kinase originally identified as a regulator for glycogen synthase production. In response to insulin, autophosphorylation of the insulin receptor occurs to provide a binding site for insulin receptor substrate-1 (IRS-1) and cause its activation. Eventually, activated IRS-1 recruits phosphatidylinositol-3-kinase (PI3K) to activate downstream molecular targets. The activation of protein kinase B (AKT) phosphorylates inhibits GSK3β to activate glycogen synthase, leading to production of glycogen [4]. Despite GSK3β traditionally acting as a negative regulator of glycogen synthesis, it has been identified as a central point of convergence for multiple intracellular signalling pathways involved in various physiological processes [5]. In the control of osteogenesis, the inhibition of GSK3β enhances osteoblastic differentiation through accumulation and nuclear translocation of β-catenin [6]. However, the effects of GSK3β on osteoclast formation and activity remain largely unidentified.

The current review highlights the specific role of GSK3β in osteoclast formation and its activity. Both in vivo and in vitro studies reporting the phosphorylation status of GSK3β, bone resorption, and osteoclast-specific markers were summarised. The efficiency of GSK3β on maintaining bone health was also collated. It is hypothesised that GSK3β has an essential role in osteoclast differentiation and formation by serving as an intermediate product, integrating numerous signal transduction pathways.

## 2. Literature Search

**Figure 1:**
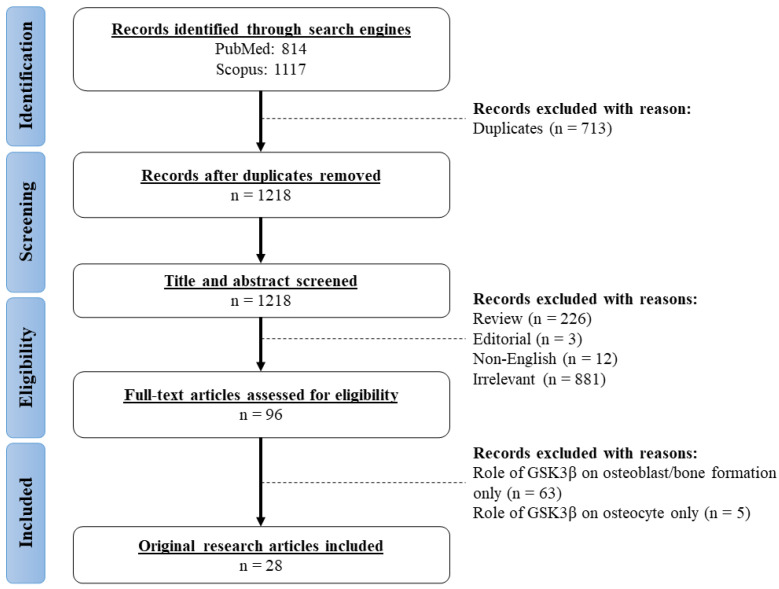
Workflow for literature search.

The research questions of current review were: what is the role of GSK3β and its underlying mechanisms in orchestrating osteoclastogenesis and bone resorption? To identify relevant studies, a comprehensive literature search was conducted across in two electronic databases (PubMed and Scopus) in January 2024 using the keyword string: (“glycogen synthase kinase 3” OR GSK3) AND (bone OR osteoporosis OR fracture OR osteoblast OR osteoclast OR osteocyte). All available records from the inception of the databases were gathered. A total of 814 records were retrieved from PubMed database, whereas 1117 records were obtained from the Scopus database. After removing duplicates (*n* = 713), preliminary screening of the title and abstract were performed to identify and exclude reviews (*n* = 226), editorials (*n* = 3), non-English (*n* = 12), and irrelevant articles (*n* = 881). Subsequently, full-text articles were screened for eligibility based on the inclusion and exclusion criteria. All original research articles that reported on the phosphorylation status of GSK3β and parameters associated with osteoclastic bone resorption in vivo and in vitro were included. Additionally, studies illustrating the effects of GSK3β inhibitors/knockdown on osteoclastogenesis and bone resorption were included. Studies were excluded if the role of GSK3β on osteogenesis, bone formation, osteoblasts, and osteocytes were reported. A total of 28 articles adhering to the predefined inclusion criteria were incorporated into this review. Although the osteogenic and mechanosensory role of GSK3β in osteoblasts and osteocytes was not discussed in the current review, they are used as keywords for the literature search to avoid overlooking studies with various bone parameters (Figure 1).

## 3. In Vivo Evidence on the Role of GSK3β on Bone Resorption

The in vivo experimental evidence on the involvement of GSK3β in bone resorption has been accumulated (Table 1). Postmenopausal osteoporosis is the most common form of osteoporosis. Several studies utilised ovariectomised rodent model of bone loss to mimic the clinical manifestations of postmenopausal osteoporosis. The ovariectomised rodents displayed higher Oc.N and a reduced OPG/RANKL ratio, resulting in low quality of trabecular bone microstructure [7,8]. However, the level of GSK3β was not affected in the ovariectomised rodents [7]. Treatment with penicopeptide A isolated from deep sea-derived fungus improved bone quality in ovariectomised mice, but did not have any effect and Oc.N and expression of GSK3β [7]. AZD1390 is a brain penetrant ataxia telangiectasia mutant (ATM) kinase inhibitor that blocks ATM-dependent signalling and repair of deoxyribonucleic acid (DNA) double-strand breaks. For parameters related to osteoclastogenesis, treatment of AZD1390 reduced Oc.N and cathepsin K (CTSK) expression level in the ovariectomised mice [9]. Oral administration of imperatorin reduced TRAP staining indicating low osteoclast activity in the ovariectomised rats [10]. One of the downstream signalling cascades within the RANK/RANKL/OPG pathway is the activation of PI3K, which induces a series of kinase activities through phosphoinositide-dependent kinase 1 (PDK1), AKT, and GSK3β [11]. Thus, PDK1 is a downstream effector of PI3K responsible for AKT and GSK3β phosphorylation, indicating that PDK1 is a pivotal molecule for committing the monocytic cells to osteoclast lineage. A study conducted by Xiao et al. investigated the role of PDK1 in osteoclasts using PDK1-knockout mice subjected to ovariectomy. The findings showed that PDK1 deficiency protected the animal from bone loss induced by ovariectomy. The Oc.N, Oc.S, and circulating bone resorption markers [including C-terminal crosslinking telopeptide of type I collagen (CTX-1) and TRAP] were reduced, leading to the improvement in lumbar bone microarchitecture [12].

Hypercaloric intake, such as consuming a diet high in fat and carbohydrate, was associated with deterioration of bone quality and strength [13,14,15,16,17]. A study by Bu and co-researchers showed that mice fed on a high-fat diet for 8 weeks to induce obesity had low bone mass with higher TRAP and RANKL expression but lower levels of OPG. The obese mice also exhibited oxidative damage, evidenced by a lowering of superoxide dismutase (SOD), glutathione peroxidase (GPX), and catalase (CAT) (the antioxidant capacity biomarkers) but increasing of malondialdehyde (MDA) (a marker of lipid peroxidation). The protein level of phosphorylated GSK3β, nuclear factor-erythroid 2-related factor 2 (NRF2), and heme-oxygenase 1 (HO-1) were found to be lower after feeding with a high-fat diet. The supplementation of whey protein hydrolysate in a high-fat diet as intervention preserved bone mass, improved bone biomechanical properties, and attenuated oxidative damage via activating the GSK3β/NRF2/HO-1 signalling pathway [18]. A similar mechanism of action was observed in the obesity-prone rats fed with a high-fat diet. The levels of TRAP, CTSK, RANKL/OPG, matrix metalloproteinase-9 (MMP-9), TRAF6, urinary hydroxyproline, and urinary calcium were increased in the obesity-prone rats after consuming a high-fat diet, which was not seen in the obesity-resistant rats. Moreover, the mitochondrial reactive oxygen species (ROS) was raised in the obesity-prone rats as compared to the obesity-resistant rats. The intervention using 4% sodium butyrate ameliorated the high-fat diet-altered changes for osteoclastogenesis-related parameters and oxidative damage. The signalling molecules involved were reduced phosphorylation of GSK3β as well as downregulation of downstream genes of the NRF2 signalling pathway, including nicotinamide-adenine dinucleotide phosphate hydrogen quinone dehydrogenase 1 (NQO1) and HO-1 in high-fat diet fed obesity-prone rats. These alterations were reversed when sodium butyrate was administered [19].

Certain drugs or medicines are deleterious on bone health. Glucocorticoid-induced osteoporosis is the most common cause of iatrogenic osteoporosis. The increases in Oc.N, osteoclast surface (Oc.S), RANKL expression, and the reduction of OPG expression were observed in glucocorticoid-induced osteoporotic animals. Various treatments, including luteolin, ferulic acid, and 6-bromoindirubin-3′-oxim, reversed glucocorticoid-induced changes in osteoclastic bone parameters [20,21,22]. In another study, the expression of RANKL was increased in streptozotocin (STZ)-induced diabetic rats with osteoporosis, whereby lower trabecular bone and bone mass were detected. The total GSK3β expression was upregulated in the diabetic and osteoporotic animal model [23].

The risk of skeletal fracture is higher in patients diagnosed with chronic kidney disease (CKD) as compared to healthy individuals. Tatsumoto and colleagues determined the effects of GSK3β inhibition on bone volume in mice with CKD. The animal models used in this study include normal and GSK3β heterozygous knockout (GSK3β^+/−^) mice fed with a 0.2% adenine diet to induce CKD. The findings of this study indicated that serum levels of PTH and TRAP were higher in the adenine-induced CKD mice as compared to the normal mice. Conversely, improvement in trabecular bone volume was seen in the adenine-diet-fed GSK3β^+/−^ mice. These results suggested the beneficial effects of GSK3β blocking on bone quality, thus reducing fracture risk in patients with CKD [24].

Titanium is a chemical element with an atomic number of 22. It connects well with bone and thus is widely used for hip joint replacements and tooth implants. Nonetheless, one major limitation is the sustained release of titanium particles at the implant site, which results in inhibition of osteoblastic activities, stimulation of osteoclast differentiation, aggravation of inflammatory response, and oxidative stress [25]. In a murine clavarial model of titanium-induced bone destruction, histological analysis showed that the eroded surface (ES), TRAP-positive multinucleated cells, and Oc.S were increased [26]. Lipopolysaccharides, a surface membrane component of gram-negative bacteria, is another potent inducer of bone resorption, upregulating osteoclast differentiation and its activities [27]. In in vivo studies, the group receiving LPS displayed a higher number of TRAP-positive osteoclasts and expression of osteoclastogenic markers [including RANKL, NFATc1, Fos proto-oncogene (c-Fos), MMP-9, CTSK, and TRAF6], as well as a higher level of inflammatory markers [interleukin-1 beta (IL-1β) and interleukin-6 (IL-6)]. The signalling molecules involved were activation of TRAF6 expression, phosphorylation of nuclear factor-kappa B (NF-κB), and degradation of IκBα (a NF-κB inhibitory protein) [28,29]. RANKL, as an important regulator of osteoclast formation and activation in the RANK/RANKL/OPG pathway, can be used to establish an osteoporotic animal model. In RANKL-induced osteoporotic mice, Shin et al. demonstrated that high bone erosion and osteoclast number were observed. These alterations were prevented by treatment of Gö6976, a PKC inhibitor [30].

This section summarises the in vivo evidence on the role of GSK3β on bone resorption. Several observations can be concluded based on the aforementioned studies. The GSK3β level was unaltered in ovariectomised rodents, mimicking the pathophysiology of postmenopausal osteoporosis. Similarly, the GSK3β level was unaffected after pharmacological treatment. It could be theorised that the occurrence of bone loss might be attributed to the direct action of oestrogen deficiency on OPG and RANKL production in osteoblasts, or via other signalling molecules in the RANK/RANKL/OPG pathway. In contrast, the level of phosphorylated GSK3β was reduced (favouring GSK3β activation) in obesity-related and glucocorticoid-induced deterioration of bone health. The mechanism of action involved was mainly the induction of oxidative stress through suppression of the NRF2 signalling pathway. The expression of GSK3β (activation of GSK3β) was also increased in STZ-induced diabetic rats with osteoporosis, which was associated with the increased inflammatory response and impaired glycaemic control in this model. Treatment using various pharmacological bone-protecting agents caused the phosphorylation and inhibition of GSK3β, leading to better bone health. However, only one study reported that GSK3β gene expression (activity) was lowered in glucocorticoid-induced osteoporosis, which was then increased following improvement of bone parameters after treatment with ferulic acid [21]. The discrepancy could be due to the fact that the total gene GSK3β expression was measured, thus the phosphorylation status of GSK3β was unresolved. No result on GSK3β expression was reported in the titanium-induced osteolysis, or LPS- and RANKL-induced osteoporosis. In short, it is recommended that the expression of both total and phosphorylated GSK3β should be evaluated in bone tissues to provide a better understanding of the role of the activation or inhibition of GSK3β in governing osteoclastic activity in animals.

**Table 1:** The effects of GSK3β on osteoclastogenesis in animal studies.

Animal Model	Intervention and Dose	Findings	Changes in GSK3β	Reference
Ovariectomised rats	-	BV/TV: ↓, Tb.N: ↓, Tb.Th: ↔, Tb.Sp: ↑, cortical parameters: ↔, MS: ↑, MAR: ↓, BFR: ↑, load: ↔, work to failure: ↔, stiffness: ↔, Oc.N: ↑, OPG/RANKL: ↓	-	[8]
Ovariectomised mice	-	BV/TV: ↓, Tb.N: ↓, Tb.Th: ↔, Tb.Sp: ↑, Ct.Th: ↓, MS: ↓, MAR: ↓, BFR: ↓, Oc.N: ↑, GSK3β: ↔	No difference in GSK3β protein expression among normal and oestrogen-deficient rats	[7]
	Penicopeptide A (10 mg/kg, i.v., once every 2 days for 6 weeks)	BV/TV: ↑, Tb.N: ↑, Tb.Th: ↔, Tb.Sp: ↔, Ct.Th: ↔, MS: ↑, MAR: ↑, BFR: ↑, Oc.N: ↔, GSK3β: ↔		
Ovariectomised mice	AZD1390 (0.1–1 mg/kg)	BV/TV: ↑, Tb.N: ↑, Tb.Sp: ↓, Oc.N: ↓, CTSK: ↓	-	[9]
Ovariectomised rats	Imperatorin (20 mg/kg)	BV/TV: ↑, Tb.N: ↑, Tb.Sp: ↓, TRAP: ↓	-	[10]
PDK1-knockout and ovariectomised mice	-	BV/TV: ↑, Tb.N: ↑, Tb.Th: ↑, Conn.D: ↑, Tb.Sp: ↓, SMI: ↓, MAR: ↔, Oc.N: ↓, Oc.S: ↓, CTX-1: ↓, TRAP: ↓	-	[12]
High-fat-diet-fed mice	-	BMD: ↓, BV/TV: ↓, Tb.N: ↓, Tb.Th: ↓, Tb.Sp: ↑, calcium: ↓, phosphorus: ↓, load: ↓, stiffness: ↓, TRAP: ↑, OPG: ↓, RANKL: ↑, SOD: ↓, GPX: ↓, CAT: ↓, MDA: ↑, p-GSK3β: ↓, NRF2: ↓, HO-1: ↓	Phosphorylation of GSK3β was inhibited (GSK3β was activated) in high-fat diet-induced osteoporosis, which was reversed after intervention using whey protein	[18]
	Whey protein hydrolysate (4% diet)	BMD: ↑, BV/TV: ↑, Tb.N: ↑, Tb.Th: ↔, Tb.Sp: ↓, calcium: ↑, phosphorus: ↑, load: ↔, stiffness: ↑, TRAP: ↓, OPG: ↔, RANKL: ↓, SOD: ↑, GPX: ↑, CAT: ↑, MDA: ↓, p-GSK3β: ↑, NRF2: ↑, HO-1: ↑		
Obesity-prone rats fed with high-fat diet	-	BV/TV: ↓, Tb.N: ↔, Tb.Th: ↓, Tb.Sp: ↔, SMI: ↔, femoral calcium: ↓, vitamin D: ↑, calcitonin: ↔, urinary hydroxyproline: ↑, urinary calcium: ↑, TRAP: ↑, CTSK: ↑, RANKL/OPG: ↑, MMP-9: ↑, NFATc1: ↔, TRAF6: ↑, ROS: ↑, GPX: ↔, SOD: ↔, p-GSK3β: ↓, NRF2: ↓, NQO-1: ↓, HO-1: ↓	Phosphorylation of GSK3β was inhibited (GSK3β was activated) in obesity-prone rats, which was reversed after intervention using sodium butyrate	[19]
	Sodium butyrate (4%)	BV/TV: ↔, Tb.N: ↔, Tb.Th: ↔, Tb.Sp: ↔, SMI: ↔, femoral calcium: ↑, vitamin D: ↓, calcitonin: ↔, urinary hydroxyproline: ↓, urinary calcium: ↓, TRAP: ↓, CTSK: ↓, RANKL/OPG: ↓, MMP-9: ↓, NFATc1: ↔, TRAF6: ↓, ROS: ↓, GPX: ↔, SOD: ↔, p-GSK3β: ↑, NRF2: ↑, NQO-1: ↑, HO-1: ↑		
Dexamethasone-induced osteoporotic rats	-	BMC: ↓, BMD: ↓, BS: ↓, BV/TV: ↓, total mineral content: ↓, Tb.Th: ↓, Tb.N: ↓, Tb.Sp: ↑, SMI: ↑, load: ↓, bending capacity: ↓, rigidity: ↓, elasticity: ↓, OPG: ↓, RANKL: ↑, LDH: ↑, SOD: ↓, GSH: ↓, p-GSK3β: ↓	Phosphorylation of GSK3β was inhibited (GSK3β was activated) in dexamethasone-induced osteoporosis, which was reversed after intervention using luteolin	[20]
	Luteolin (25–100 mg/kg)	BMC: ↑, BMD: ↑, BS: ↑, BV/TV: ↑, total mineral content: ↑, Tb.Th: ↑, Tb.N: ↑, Tb.Sp: ↓, SMI: ↓, load: ↑, bending capacity: ↑, rigidity: ↑, elasticity: ↑, OPG: ↑, RANKL: ↓, LDH: ↓, SOD: ↑, GSH: ↑, p-GSK3β: ↑		
Dexamethasone-induced osteoporotic rats	-	BMD: ↓, biomechanical force: ↔, Ct.Th: ↓, Ct.Ar: ↓, Tb.Th: ↓, Tb.N: ↓, diaphysis thickness: ↓, bone marrow cavity: ↓, OPG: ↓, RANKL: ↑, OPG/RANKL: ↓, GSK3β: ↓	GSK3β gene expression was lowered in dexamethasone-induced osteoporosis, which was raised after intervention using ferulic acid	[21]
	Ferulic acid (50 and 100 mg/kg)	BMD: ↑, biomechanical force: ↑, Ct.Th: ↑, Ct.Ar: ↑, Tb.Th: ↑, Tb.N: ↑, diaphysis thickness: ↑, osteocyte number: ↑, bone marrow cavity: ↑, calcium: ↑, phosphorus: ↑, OPG: ↑, RANKL: ↓, OPG/RANKL: ↑, GSK3β: ↑		
Methylprednisolone-induced osteoporotic rats	-	BMC: ↓, BMD: ↓, load: ↓, elasticity: ↓, BV/TV: ↓, Tb.N: ↓, Oc.N: ↑, Oc.S: ↑, dLS: ↓, MAR: ↓, BFR: ↓, p-GSK3β: ↓	Phosphorylation of GSK3β was inhibited (GSK3β was activated) in methylprednisolone-induced osteoporosis, which was reversed after intervention using 6-bromoindirubin-3′-oxim	[22]
	6-bromoindirubin-3′-oxim (1, 10, and 50 µM)	BMC: ↑, BMD: ↑, load: ↑, elasticity: ↑, BV/TV: ↑, Tb.N: ↑, Oc.N: ↓, Oc.S: ↓, dLS: ↑, MAR: ↑, BFR: ↑, p-GSK3β: ↑		
STZ-induced diabetic rats	-	Trabecular bone: ↓, bone mass: ↓, RANKL: ↑, GSK3β: ↑, p-p38 MAPK: ↑, TNF-α: ↑, insulin: ↓	GSK3β was activated in STZ-induced osteoporosis	[23]
Adenine-induced CKD mice	-	BV/TV: ↔, Tb.N: ↔, Tb.Th: ↔, Tb.Sp: ↔, calcium: ↔, phosphate: ↑, PTH: ↑, TRAP: ↑	-	[24]
Adenine-diet-fed GSK3β^+/−^ mice	-	BV/TV: ↑, Tb.N: ↔, Tb.Th: ↑, Tb.Sp: ↔, calcium: ↔, phosphate: ↔, PTH: ↔, TRAP: ↔		
Titanium-stimulated calvariae osteolysis mice	-	BMD: ↓, BV/TV: ↓, bone thickness: ↓, ES: ↑, TRAP-positive cells: ↑, Oc.S: ↑	-	[26]
LPS-induced osteoporotic mice	-	BMD: ↓, BV/TV: ↓, Tb.N: ↓, Tb.Th: ↓, Tb.Sp: ↑, TRAP-positive cells: ↑	-	[28]
	Sec-O-glucosylhamaudol (10 mg/kg, i.p., every other day for 8 days)	BMD: ↑, BV/TV: ↑, Tb.N: ↑, Tb.Th: ↑, Tb.Sp: ↓, TRAP-positive cells: ↓		
LPS-induced osteoporotic mice	-	Total mineral content: ↓, BV/TV: ↓, Tb.N: ↓, Tb.Sp: ↑, SMI: ↑, TRAP: ↑, RANK: ↑, RANKL: ↑, IL-1β: ↑, IL-6: ↑, Oc.N: ↑, NFATc1: ↑, c-Fos: ↑, MMP-9: ↑, CTSK: ↑, TRAF6: ↑, p-p65: ↑, IκBα: ↓	-	[29]
	Monotropein (40 and 80 mg/kg)	Total mineral content: ↑, BV/TV: ↑, Tb.N: ↑, Tb.Sp: ↓, SMI: ↓, TRAP: ↓, RANK: ↓, RANKL: ↔, IL-1β: ↓, IL-6: ↓, Oc.N: ↓, NFATc1: ↓, c-Fos: ↓, MMP-9: ↓, CTSK: ↓, TRAF6: ↓, p-p65: ↓, IκBα: ↑		
RANKL-induced osteoporotic mice	-	ES: ↑, Oc.N: ↑	-	[30]
	PKC inhibitors, Gö6976 (500 nM)	ES: ↓, Oc.N: ↓		

Abbreviations: BMC: bone mineral content, BMD: bone mineral density, BFR: bone formation rate, BS: bone surface, BV/TV: bone volume/total volume, CAT: catalase, c-Fos: Fos proto-oncogene, Conn.D: connectivity density, Ct.Ar: cortical area, CTSK: cathepsin K, Ct.Th: cortical thickness, CTX-1: C-terminal crosslinking telopeptide of type I collagen, dLS: double-labelled surface, ES: eroded surface, GPX: glutathione peroxidase, GSH: reduced glutathione, GSK3β: glycogen synthase kinase-3 beta, HO-1: heme-oxygenase 1, IL-1β: interleukin-1 beta, IL-6: interleukin-6, IκBα: inhibitor of nuclear factor-kappa B, LDH: lactate dehydrogenase, MAR: mineral apposition rate, MDA: malondialdehyde, MMP-9: matrix metalloproteinase-9, MS: mineralising surface, NFATc1: nuclear factor of activated T-cells cytoplasmic 1, NQO-1: NADPH quinone dehydrogenase 1, NRF2: nuclear factor-erythroid 2-related factor 2, Oc.N: osteoclast number, Oc.S: osteoclast surface, OPG: osteoprotegerin, p-GSK3β: phosphorylated glycogen synthase kinase-3 beta, p-p38 MAPK: phosphorylated p38 mitogen-activated protein kinase, p-p65: phosphorylated nuclear factor-kappa B p65, PTH: parathyroid hormone, RANKL: receptor activator of nuclear factor-kappa B ligand, ROS: reactive oxygen species, SMI: structure model index, SOD: superoxide dismutase, Tb.N: trabecular number, Tb.Sp: trabecular separation, Tb.Th: trabecular thickness, TNF-α: tumour necrosis factor-alpha, TRAF6: tumour necrosis factor receptor-associated factor 6, TRAP: tartrate-resistant acid phosphatase, ↑: increase/upregulate, ↓: decrease/downregulate, ↔: no change.

## 4. In Vitro Evidence on Molecular Mechanism Underlying the Action of GSK3β in Regulating Osteoclastogenesis

**Figure 2:**
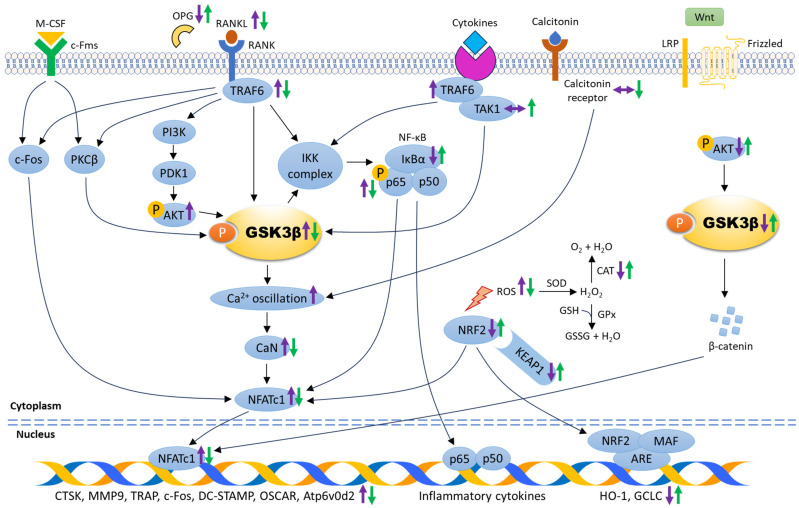
The mechanism of action underlying the role of GSK3β in regulating osteoclastogenesis. The molecular changes during osteoclastogenesis are depicted by purple arrows. The expression of signalling molecules associated with the suppression of osteoclast differentiation and maturation are indicated by green arrows. Abbreviations: ARE: antioxidant response element, Atp6v0d2: vacuolar-type proton ATPase subunit d2, Ca^2+^: calcium, CaN: calcineurin, CAT: catalase, c-Fms: colony-stimulating factor-1 receptor, c-Fos: Fos proto-oncogene, CTSK: cathepsin K, DC-STAMP: dendritic cell–specific transmembrane protein, GCLC: glutamate-cysteine ligase, GPx: glutathione peroxidase, GSH: reduced glutathione, GSSG: glutathione disulfide, HO-1: heme-oxygenase, H_2_O: water, H_2_O_2_: hydrogen peroxide, IκBα: inhibitor of nuclear factor-kappa B, IKK complax: inhibitory nuclear factor-kappa B kinase complex, KEAP1: Kelch-like ECH-associated protein 1, LRP: low-density lipoprotein receptor-related protein, MAF: small musculoaponeurotic fibrosarcoma protein, M-CSF: macrophage colony-stimulating factor, MMP9: matrix metalloproteinase 9, NFATc1: nuclear factor of activated T-cells cytoplasmic 1, NRF2: nuclear factor-erythroid 2-related factor 2, NF-κB: nuclear factor-kappa B, OPG: osteoprotegerin, OSCAR: osteoclast-associated receptor, O_2_: oxygen, PDK1: phosphoinositide-dependent kinase 1, PI3K: phosphatidylinositol-3-kinase, PKCβ: protein kinase C beta, p-AKT: phosphorylated protein kinase B, p-GSK3β: phosphorylated glycogen synthase kinase-3 beta, p-p65: phosphorylated nuclear factor-kappa B p65, RANK: receptor activator of nuclear factor-kappa B, RANKL: receptor activator of nuclear factor-kappa B ligand, ROS: reactive oxygen species, SOD: superoxide dismutase, TAK1: transforming growth factor beta-activated kinase 1, TRAF6: tumour necrosis factor receptor-associated factor 6, TRAP: tartrate-resistant acid phosphatase, ↑: increase/upregulate, ↓: decrease/downregulate, ↔: no change.

The role of GSK3β in cell differentiation, proliferation, fusion, and activation of osteoclasts was further scrutinised in in vitro studies using bone-marrow-derived monocytes or macrophages (BMMs) and murine macrophages (RAW264.7) (Table 2). These cells can be stimulated by M-CSF and/or RANKL to differentiate into mature osteoclasts [31]. Osteoclasts are giant multinucleated cells responsible for bone degradation to initiate normal bone remodelling. During osteoclastogenesis, the presence of M-CSF- and RANKL-induced calcium (Ca^2+^) oscillation (regenerative discharges of stored calcium) are required for NFATc1 activation and expression. A study by Jang et al. demonstrated that GSK3β was phosphorylated after M-CSF and RANKL treatment, suggesting that inactivation of GSK3β was crucial for osteoclast differentiation. High expression of phosphorylated GSK3β was detected as early as day two after RANKL stimulation without affecting total GSK3β level during osteoclastogenesis. The researchers further confirmed the role of GSK3β on osteoclastogenesis using RANKL-stimulated BMMs subjected to retrovirus expressing wild-type GSK3β, constitutively active GSK3β mutant, catalytically inactive GSK3β mutant, and GSK3β knockdown using small interfering RNA (siRNA). The number of TRAP-positive multinucleated cells was increased and Ca^2+^ oscillation was evident in BMMs infected with retrovirus expressing catalytically inactive GSK3β mutant, and BMMs subjected to GSK3β knockdown. Conversely, a decreased number of TRAP-positive multinucleated cells and impaired Ca^2+^ oscillation were seen in BMMs expressing wild-type GSK3β and the constitutively active GSK3β mutant [32]. With the evidence of the potential role of GSK3β in osteoclast formation and activity, extensive research has been conducted to provide a fundamental understanding of the signal transduction between GSK3β and osteoclastogenesis, including the modulation through RANK/RANKL/OPG, PI3K/AKT, NF-κB, NRF2/Kelch-like ECH-associated protein 1 (KEAP1), canonical Wnt/β-catenin, and PKC signalling pathways (Figure 2).

### 4.1. RANK/RANKL/OPG Pathway

The RANKL, RANK, and OPG are three main components that regulate osteoclast differentiation and maturation. RANKL [also known as osteoprotegerin ligand (OPGL), osteoclast differentiation factor (ODF), or TNF-related activation-induced cytokine (TRANCE)] is a type II transmembrane protein secreted by osteoblast. RANK is a receptor for RANKL which is expressed on the surface of osteoclast progenitors and mature osteoclasts. OPG is produced by osteoblast, preventing the RANK–RANKL interaction [33]. In other words, osteoblast influences osteoclast formation and its activity via the expression of OPG and RANKL. Hence, the effects of GSK3β on OPG and RANKL expression were evaluated using murine pre-osteoblastic (MC3T3-E1) cells (Table 3).

Using MC3T3-E1 cells, two recent studies have demonstrated that dexamethasone decreased OPG levels but increased RANKL expression as compared to the normal control. The reduced OPG/RANKL ratio caused by dexamethasone was improved by the treatment of luteolin (0.1–0.2 μM) and ferulic acid (0.5–3 μM). The phosphorylated GSK3β was declined in the dexamethasone group, which was increased upon receiving luteolin and ferulic acid [20,21]. A similar trend for the OPG/RANKL ratio was detected in another in vitro model utilising MC3T3-E1 cells stressed with titanium particles. Titanium particles induced RANKL expression and decreased OPG levels when GSK3β phosphorylation was inhibited as compared to the control group [26]. The ligation of RANKL to its receptor (RANK) leads to the recruitment and binding of TRAF6 to the cytoplasmic domain of RANK. Several downstream signalling pathways are consecutively activated to promote osteoclast formation, activation, and survival upon the RANK/TRAF6 interaction, which consists of the PI3K/AKT, NF-κB, MAPK/AP-1, and calcineurin/NFATc1.

### 4.2. PI3K/AKT Signalling Pathway

The RANKL–RANK interaction triggers the activation of PI3K. Activated PI3K further promotes the catalytic conversion of phosphatidylinositol-4,5-bisphosphate (PIP_2_) to phosphatidylinositol-3,4,5-trisphosphate (PIP_3_). Phosphoinositide-dependent kinase 1 (PDK1) is the downstream serine/threonine protein kinase recruited by PIP_3_ to phosphorylate and activate AKT. Following that, GSK3β is phosphorylated and inhibited, serving as a negative regulator to increase NFATc1 nuclear transportation and transcriptional activity [4,34].

In the presence of M-CSF and RANKL, the formation of TRAP-positive multinucleated cells, an F-actin ring, and a resorption pit were significantly induced in BMMs. The expression of osteoclastogenic target genes [such as c-Fos, CTSK, TRAP, matrix metalloproteinase-9 (MMP9), dendritic cell-specific transmembrane protein (DC-STAMP), and Atp6v0d2] were upregulated upon RANKL stimulation. The p-AKT/AKT and p-GSK3β/GSK3β ratio were found to be increased during osteoclastogenesis. Incubation of M-CSF- and RANKL-stimulated BMMs with natural products (sec-O-glucosylhamaudol and purple tea water extract) resulted in the reduction of AKT and GSK3β phosphorylation, causing the inhibition of mature osteoclast formation [28,35]. A similar trend in the phosphorylation status of AKT and GSK3β was noted in LPS-treated BMMs [29]. To further confirm that the regulation of GSK3β can be mediated through the PI3K/AKT signalling pathway, Chen et al. attempted to investigate the effects of LY3023414 [a novel oral PI3K/mammalian target of rapamycin (mTOR) dual inhibitor] on osteoclastogenesis. LY3023414 attenuated osteoclast formation as well as PI3K/AKT/GSK3β-dependent osteoclast-specific gene expression (TRAP, calcitonin receptor, CTSK, and NFATc1) in M-CSF- and RANKL-stimulated BMMs. The phosphorylation of AKT and GSK3β was suppressed by LY3023414 treatment [36]. Likewise, the osteoclast cell formation was reduced and expression of osteoclast-specific genes, including NFATc1 and OSCAR, was downregulated in BMMs after treatment with LY294002 (another specific inhibitor of PI3K). In the same study, the researchers proved that AKT overexpression caused GSK3β phosphorylation (inactivation), leading to nuclear localisation of NFATc1 and osteoclastic-specific gene expression in osteoclasts. Upon GSK3β overexpression, the NFATc1 level was reduced in osteoclasts [37].

Guanine nucleotide-binding protein subunit α13 (Gα13) is a negative regulator of osteoclastogenesis. In a study by Wu et al., Gα13 deficiency increased phosphorylation of AKT and GSK3β to trigger a drastic increase in osteoclast number and activity. AKT inhibition rescued the hyperactivation of osteoclasts, which resulted from Gα13 deficiency. In addition, Gα13 overexpression inhibited AKT and activated GSK3β, providing inhibitory effects on osteoclastogenesis [38]. In vitro, AZD1390 halted osteoclastogenesis by suppressing the PI3K/AKT signalling cascade. Protein expression of p-AKT and p-GSK3β were decreased after treatment with AZD1390 [9]. PDK1 serves as the downstream effector of the PI3K essential for AKT phosphorylation. The absence of PDK1 was associated with impaired osteoclast formation and bone resorption, which ultimately delayed the fracture repair and healing processes [39]. In BMMs harvested from PDK1-knockout mice, the Oc.N and expression of osteoclast-related genes were lowered under the stimulation of M-CSF and RANKL. The AKT and GSK3β phosphorylation was noticeably reduced in BMMs of the PDK1-knockout mice as compared to the control mice [12]. Microtubule actin crosslinking factor 1 (MACF1) is a critical spectraplakin (giant multifunctional cytoskeletal proteins that connect the cytoskeletal filaments and master their coordination) that is ubiquitously expressed in various tissues such as brain, spinal cord, heart, kidney, lung, nerve, skin, and skeletal muscles [40,41]. The silencing of MACF1 resulted in inhibition of multinucleated osteoclast formation and osteoclastogenic gene expression in RANKL-induced RAW264.7 cells. Knockdown of MACF1 also abrogated the AKT and GSK3β phosphorylation in RANKL-induced osteoclastogenesis. In the presence of an AKT activator, the inhibition of osteoclast differentiation caused by MACF1 knockdown was rescued [42].

Taken together, this research provides homogenous evidence implying that the activation of the PI3K/AKT signalling cascade causes the inhibition of GSK3β, leading to osteoclastogenesis.

### 4.3. NF-κB Signalling Pathway

Chronic inflammation is the hallmark of bone loss. Inflammatory mediators disrupt bone homeostasis by increasing bone resorption and decreasing bone formation. NF-κB is a transcription factor with a well-recognised function in encoding pro-inflammatory cytokines in innate immune cells, thus orchestrating inflammatory response in the body. NF-κB is inactive and sequestered in the cytoplasm under basal conditions, consisting of inhibitory NF-κB protein (IκBα) that binds to p65/p50 heterodimer. With the presence of RANKL or pro-inflammatory stimuli, TRAF6 and transforming growth factor beta-activated kinase 1 (TAK1) are two key upstream signalling molecules that activate the inhibitory NF-κB kinase (IKK) complex. This complex induces phosphorylation and degradation of IκBα, thus releasing p65/p50 heterodimer to be translocated into the nucleus. Following this, the transcription of c-Fos and NFATc1 essential for osteoclast differentiation and maturation occurs [43,44]. GSK3β is a double-edged sword, causing both stimulatory and inhibitory regulation in the NF-κB signalling cascade, depending on the cell type and physiological state of the cell. For instance, GSK3β stimulates NF-κB activity in tumour cells or cells with increased inflammatory response. Conversely, the constitutively active GSK3β in a physiological state inhibits the IKK complex and nuclear translocation of p65/p50 subunits of NF-κB [45].

Sujitha and co-researchers reported higher expression of NFATc1, MMP9, CTSK, TRAP, and calcineurin (CaN) in BMMs exposed to M-CSF and RANKL. With the observations of increased osteoclastogenesis, GSK3β was phosphorylated but expression of TAK1 was unchanged. The incubation of M-CSF- and RANKL-induced BMMs with berberine-coated mannosylated liposomes suppressed osteoclast differentiation with increased expression of TAK1 [46]. The number of TRAP-positive multinucleated cells, formation of the F-actin ring, and expression of genes involved in osteoclast formation were raised in LPS-treated BMMs. The underlying signalling molecules involved were upregulation of TRAF6, phosphorylation (activation) of p65 NF-κB, degradation of IκBα, and nuclear export of p65 in osteoclasts. The inhibition of osteoclastogenesis by monotropein was modulated through inactivation of the NF-κB signalling pathway. However, researchers found that the GSK3β activity was inhibited when NF-κB was activated in LPS-treated BMMs. The relief of the inflammatory response in LPS-induced osteoclastogenesis by monotropein was mediated through suppression of the NF-κB pathway and reduced phosphorylation of GSK3β (GSK3β was activated) [29]. Two possible assumptions can be made for such observations. Firstly, LPS and RANKL may have a direct action on NF-κB signalling molecules for the induction of inflammation during osteoclast formation. Secondly, both activation and inhibition of GSK3β may occur in parallel via the NF-κB and PI3K/AKT signalling. The inhibition of GSK3β activity observed in the study by Zhang et al. may be the net outcome from the activation of both pathways.

### 4.4. NRF2/KEAP1 Signalling Pathway

ROS are the intracellular mediators synthesised in response to RANKL/RANK interaction to promote bone resorption, which is neutralised by the antioxidant defence system. The perturbation in the delicate balance between ROS level and antioxidant capacity, characterised by high ROS level and low antioxidant level, further enhances osteoclastic activity and structural deterioration in bone. Apart from the well-known action of the NRF2/KEAP1 signalling pathway as a regulator for antioxidative enzyme transcription providing cellular resistance to oxidative stress, the increase in the NRF2 signalling molecule inhibits RANKL and NF-κB expression to impede the efficacy of NFATc1 in osteoclast differentiation and formation. During osteoclastogenesis, NRF2 expression is lowered, compromising the antioxidant defence and augmenting the transcription activity of genes associated with osteoclastogenic differentiation [47]. Theoretically, active GSK3β negatively regulates NRF2 activity via phosphorylation and, subsequently, degradation of NRF2 [48].

The presence of M-CSF and RANKL promoted the differentiation of BMMs into mature osteoclasts and their activity through regulation of the oxidative pathway and associated target genes. Specifically, the ROS level was elevated but expression of antioxidants such as NRF2, KEAP1, CAT, HO-1, and glutamate-cysteine ligase (GCLC) were downregulated during osteoclastogenesis, which was reversed following the treatment of AZD1390. In this study, the inhibition of GSK3β was more favourable in oxidative stress conditions during osteoclast differentiation and maturation [9]. Findings from this study contradict the basic knowledge about the action of GSK3β on NRF2/KEAP1 signalling, for which further validation is required.

### 4.5. Canonical Wnt/β-Catenin Signalling Pathway

Although the canonical Wnt/β-catenin signalling is the most implicated pathway for osteogenesis, Yan et al. investigated the role of this signal transduction during osteoclastogenesis. In BMMs induced by M-CSF and RANKL, the protein expression of phosphorylated GSK3β and β-catenin was lowered. Treatment of imperatorin, a bioactive compound available in traditional Chinese medicine, namely Angelica archangelica and Peucedanum praeruptorum, reduced the number of TRAP-positive multinucleated cells and decreased expression of NFATc1, c-Fos, and TRAP. The molecular mechanism underlying the anti-osteoclastogenic activities of imperatorin was mediated through activation of AKT, inhibition of GSK3β, and degradation of β-catenin in BMMs stimulated by M-CSF and RANKL [10].

### 4.6. Protein Kinase C (PKC)

Protein kinase C beta (PKCβ) plays a crucial role in osteoclast differentiation. The treatment of the PKCβ inhibitor effectively suppressed the formation of TRAP-positive multinucleated cells and NFATc1 induction in BMMs stimulated by M-CSF and RANKL, suggesting the role of PKCβ in osteoclast differentiation. To further understand the signalling link between PKCβ and GSK3β in RANKL-mediated osteoclast differentiation, PKCβ silencing was performed in BMMs using short-hairpin ribonucleic acid (shRNA) targeting PKCβ. The downregulation of PKCβ decreased GSK3β phosphorylation and osteoclastogenic gene expression [30]. These findings reiterated the protective effects of PKCβ inhibition against RANKL-induced osteoclastogenesis by activating GSK3β.

**Table 2:** The effects of GSK3β on osteoclast cells.

Type of Cells	Intervention and Concentration	Findings	Changes in GSK3β	Reference
M-CSF- and RANKL-stimulated BMMs	-	Ca^2+^ oscillation: ↑, p-GSK3β: ↑, GSK3β: ↔	GSK3β was phosphorylated (inhibited) during osteoclastogenesis	[32]
M-CSF- and RANKL-stimulated BMMs	Sec-O-glucosylhamaudol (100–200 μM)	TRAP-positive multinucleated cells: ↓, F-actin number: ↓, resorption pit: ↓, NFATc1: ↓, c-Fos: ↓, TRAP: ↓, CTSK: ↓, DC-STAMP: ↓, p-AKT: ↓, p-GSK3β: ↓	GSK3β was dephosphorylated (activated) during reduced osteoclastogenesis after intervention using Sec-O-glucosylhamaudol	[28]
M-CSF- and RANKL-stimulated BMMs	Purple tea extract	Oc.N: ↓, resorption pit: ↓, F-actin ring: ↓, NFATc1: ↓, c-Fos: ↓, DC-STAMP: ↓, CTSK: ↓, Atp6v0d2: ↓, p-AKT: ↓, p-GSK3β: ↓	GSK3β was dephosphorylated (activated) during reduced osteoclastogenesis after intervention using purple tea extract	[35]
M-CSF- and RANKL-stimulated BMMs	-	TRAP-positive multinucleated cells: ↑, resorbed area: ↑, NFATc1: ↑, MMP9: ↑, CTSK: ↑, TRAP: ↑, CaN: ↑, TAK1: ↔, p-GSK3β: ↑	GSK3β was phosphorylated (inhibited) during osteoclastogenesis, which was reversed after intervention using berberine-coated mannosylated liposomes	[46]
	Berberine-coated mannosylated liposomes	TRAP-positive multinucleated cells: ↓, resorbed area: ↓, NFATc1: ↓, MMP9: ↓, CTSK: ↓, TRAP: ↓, CaN: ↓, TAK1: ↑, p-GSK3β: ↓		
M-CSF-, RANKL-, and LPS-stimulated BMMs	-	Oc.N: ↑, TRAP: ↑, NFATc1: ↑, c-Fos: ↑, MMP9: ↑, CTSK: ↑, TRAF6: ↑, p-p65: ↑, IκBα: ↓, p-GSK3β: ↑, p-AKT: ↑	GSK3β was phosphorylated (inhibited) during osteoclastogenesis, which was reversed after intervention using monotropein	[29]
	Monotropein (0.1–10 μM)	Oc.N: ↓, TRAP: ↓, NFATc1: ↓, c-Fos: ↓, MMP9: ↓, CTSK: ↓, TRAF6: ↓, p-p65: ↓, IκBα: ↑, p-GSK3β: ↓, p-AKT: ↓		
M-CSF- and RANKL-stimulated BMMs	LY3023414 (80–160 μM)	Oc.N: ↓, TRAP: ↓, calcitonin receptor: ↓, CTSK: ↓, NFATc1: ↓, p-AKT: ↓, p-GSK3β: ↓	GSK3β was dephosphorylated (activated) during reduced osteoclastogenesis after intervention using LY3023414	[36]
M-CSF- and RANKL-stimulated BMMs	LY294002 (PI3K inhibitor)	TRAP-positive multinucleated cells: ↓, p-AKT: ↓, c-Fos: ↔, NFATc1: ↓, OSCAR: ↓	GSK3β was phosphorylated (inhibited) during osteoclastogenesis, which was reversed after GSK3β overexpression	[37]
M-CSF- and RANKL-stimulated BMMs treated with AKT overexpression	-	TRAP-positive multinucleated cells: ↑, NFATc1: ↑, OSCAR: ↑, p-GSK3β: ↑		
	LY294002 (PI3K inhibitor)	TRAP-positive multinucleated cells: ↓		
M-CSF- and RANKL-stimulated BMMs treated with GSK3β overexpression	-	TRAP-positive multinucleated cells: ↓, NFATc1: ↓		
M-CSF- and RANKL-stimulated BMMs	Gα13 overexpression	TRAP-positive multinucleated cells: ↓, resorbed area: ↓, F-actin ring: ↓, CTSK: ↓, NFATc1: ↓, p-AKT: ↓	GSK3β was phosphorylated (inhibited) by Gα13 knockdown during osteoclastogenesis	[38]
	Gα13 knockdown	p-AKT: ↑, p-GSK3β: ↑, NFATc1: ↑		
M-CSF- and RANKL-stimulated BMMs	AZD1390 (2 μM)	TRAP-positive multinucleated cells: ↓, osteoclast pseudopod formation: ↓, NFATc1: ↓, TRAP: ↓, c-Fos: ↓, Atp6v0d2: ↓, MMP9: ↓, p-AKT: ↓, p-GSK3β: ↓, ROS: ↓, NRF2: ↑, KEAP1: ↑, CAT: ↑, HO-1: ↑, GCLC: ↑	GSK3β was dephosphorylated (activated) during reduced osteoclastogenesis after intervention using AZD1390	[9]
M-CSF- and RANKL-stimulated BMMs derived from PDK1-knockout mice	-	Oc.N: ↓, resorbed area: ↓, CTSK: ↓, MMP9: ↓, NFATc1: ↓, TRAP: ↓, p-AKT: ↓, p-GSK3β: ↓	GSK3β was dephosphorylated (activated) during reduced osteoclastogenesis following PDK1 knockout	[12]
RANKL-stimulated RAW264.7 cells	MACF1 knockdown	TRAP-positive multinucleated cells: ↓, TRAP: ↓, MMP9: ↓, F-actin ring: ↓, DC-STAMP: ↓, p-AKT: ↓, p-GSK3β: ↓, NFATc1: ↓, CTSK: ↓	GSK3β was dephosphorylated (activated) during reduced osteoclastogenesis following MACF1 knockdown	[42]
M-CSF- and RANKL-stimulated BMMs	Imperatorin (75–300 μmol/L)	Osteoclast differentiation: ↓, NFATc1: ↓, c-Fos: ↓, TRAP: ↓, p-GSK3β: ↑, p-AKT: ↑, p-β-catenin: ↑	GSK3β was phosphorylated (inhibited) during reduced osteoclastogenesis after intervention using imperatorin	[10]
M-CSF- and RANKL-stimulated BMMs	PKC inhibitors Gö6976	TRAP-positive multinucleated cells: ↓, p-GSK3β: ↓, NFATc1: ↓, Atp6v0d2: ↓	GSK3β was dephosphorylated (activated) during reduced osteoclastogenesis by PKC inhibition or knockdown	[30]
	PKCβ knockdown	p-GSK3β: ↓, NFATc1: ↓		

Abbreviations: Atp6v0d2: vacuolar-type proton ATPase subunit d2, BMMs: bone-marrow-derived monocytes or macrophages, Ca^2+^: calcium, CaN: calcineurin, CAT: catalase, c-Fos: Fos proto-oncogene, CTSK: cathepsin K, DC-STAMP: dendritic cell–specific transmembrane protein, Gα13: guanine nucleotide-binding protein subunit α13, GCLC: glutamate-cysteine ligase, GSK3β: glycogen synthase kinase-3 beta, HO-1: heme-oxygenase 1, IκBα: inhibitor of nuclear factor-kappa B, KEAP1: Kelch-like ECH-associated protein 1, MACF1: microtubule actin crosslinking factor 1, M-CSF: macrophage colony-stimulating factor, MMP9: matrix metalloproteinase-9, NFATc1: nuclear factor of activated T-cells cytoplasmic 1, NRF2: nuclear factor-erythroid 2-related factor 2, OSCAR: osteoclast-associated receptor, p-AKT: phosphorylated protein kinase B, p-GSK3β: phosphorylated glycogen synthase kinase-3 beta, PKC: protein kinase C, PKCβ: protein kinase C beta, p-p65: phosphorylated nuclear factor-kappa B p65, RANKL: receptor activator of nuclear factor-kappa B ligand, ROS: reactive oxygen species, TAK1: transforming growth factor beta-activated kinase 1, TRAF6: tumour necrosis factor receptor-associated factor 6, TRAP: tartrate-resistant acid phosphatase, ↑: increase/upregulate, ↓: decrease/downregulate, ↔: no change.

**Table 3:** The effects of GSK3β on OPG and RANKL expression in osteoblast cells.

Type of Cells	Intervention and Concentration	Findings	Changes in GSK3β	Reference
Dexamethasone-induced MC3T3-E1 cells	-	OPG: ↓, RANKL: ↑, OPG/RANKL: ↓, p-GSK3β: ↓	GSK3β was dephosphorylated (activated) during low OPG/RANKL ratio in osteoblast, which was reversed after intervention using luteolin	[20]
	Luteolin (0.1–0.2 µM)	OPG: ↑, RANKL: ↓, OPG/RANKL: ↑, p-GSK3β: ↑		
Dexamethasone-induced MC3T3-E1 cells	-	OPG: ↓, RANKL: ↓, OPG/RANKL ratio: ↓, LDH: ↓, ROS: ↑, SOD: ↑, GSH: ↑, MDA: ↓, GSK3β: ↓	GSK3β expression level was lowered during low OPG/RANKL ratio in osteoblast, which was raised after intervention using ferulic acid	[21]
	Ferulic acid (0.5–3 μM)	OPG: ↑, RANKL: ↓, OPG/RANKL ratio: ↑, LDH: ↓, ROS: ↓, SOD: ↑, GSH: ↑, MDA: ↓, GSK3β: ↑		
Titanium-particle-stressed MC3T3-E1 cells	-	p-GSK3β: ↓, OPG: ↓, RANKL: ↑, RANKL/OPG ratio: ↑	GSK3β was dephosphorylated (activated) during high RANKL/OPG ratio in osteoblast	[26]

Abbreviations: GSH: reduced glutathione, GSK3β: glycogen synthase kinase-3 beta, LDH: lactate dehydrogenase, MC3T3-E1: murine pre-osteoblastic cells, MDA: malondialdehyde, OPG: osteoprotegerin, p-GSK3β: phosphorylated glycogen synthase kinase-3 beta, RANKL: receptor activator of nuclear factor-kappa B ligand, ROS: reactive oxygen species, SOD: superoxide dismutase, ↑: increase/upregulate, ↓: decrease/downregulate.

## 5. The Potential Use of GSK3β Inhibitors as Anti-Resorptive Agents

The skeletal-health-enhancing properties of lithium chloride (LiCl), a well-known GSK3 inhibitor, have been extensively reviewed [49]. Herein, the use of various GSK3 inhibitors on GSK3β phosphorylation status and osteoclast-related bone parameters in vivo (Table 4) and in vitro (Table 5) was collated.

Using healthy male mice, oral administration of LiCl (10 mg/kg) for six weeks resulted in increased OPG/RANKL ratio via GSK3β inhibition (evidenced by increased phosphorylated to total GSK3β ratio) and decreased phosphorylation of downstream target (β-catenin) [50]. In female Sprague Dawley rats, treatment with another GSK3 inhibitor (AZD2858, 20 mg/kg) for two months improved both trabecular and cortical bone health even though CTX-1 level was increased and osteoclast number (Oc.N) was unaltered [51]. In adenine-induced CKD mice, the supplementation of LiCl (150 mg/L) in drinking water for six weeks increased BV/TV and Tb.Th in the trabecular region without affecting cortical bone parameters and TRAP level [24]. In ovariectomised rats, a GSK3α/β dual inhibitor (603287-31-8, 3 mg/kg) was found to improve bone mass and strength without affecting Oc.N and OPG/RANKL ratio [8]. Recently, Amirhosseini et al. demonstrated that daily treatment of 20 mg/kg AR28 (a GSK3β inhibitor) increased trabecular BV/TV, Oc.N, and OPG expression, but there was no effect on TRAP level in rats with instability-induced osteolysis [52]. Comparably, a gavage-fed higher dose of LiCl (200 mg/kg) increased bone mineral density (BMD), BV/TV, and bone thickness while it decreased ES, Oc.N, and Oc.S in mice presented with titanium-particle-induced osteolysis [26].

In a cell culture study, heterogenous results were obtained regarding the effects of GSK3β on osteoclastogenesis. Gu et al. confirmed that the addition of LiCl reduced the RANKL/OPG ratio and caused GSK3β phosphorylation in MC3T3-E1 cells treated with titanium particles [26]. In murine osteoclast precursor (RAW-D) cells, LiCl (10 mM) and SB216763 (10 μM) suppressed osteoclastogenesis by inhibiting NFATc1 upregulation [53]. An experiment undertaken by Sujitha and Rasool pointed out that BMMs exposed to M-CSF, RANKL, and LY2090314 (a GSK3β inhibitor) treatment increased TAK1 to inhibit GSK3β, resulting in the reduction of CaN and NFATc1 expression [46]. Conversely, LiCl (5 mM) was recently found to increase the number of TRAP-positive multinucleated cells, NFATc1 translocation, and GSK3β phosphorylation in BMMs stimulated by M-CSF and RANKL [54].

**Table 4:** In vivo evidence for the protective effects of GSK3β inhibitor on bone health.

Animal Model	Intervention and Dose	Findings	Reference
Male mice	LiCl (10 mg/kg/day, oral, 6 weeks)	OPG: ↑, RANKL: ↔, OPG/RANKL: ↑, p-GSK3β/total GSK3β: ↑, p-β-catenin: ↓	[50]
Female rats	GSK3 inhibitor AZD2858 (20 mg/kg/day, 2 weeks)	BMC: ↑, BMD: ↑, BV/TV: ↑, Tb.N: ↑, Tb.Th: ↑, Ct.Ar: ↑, Ct.Th: ↑, MAR: ↑, BFR: ↑, load: ↑, stiffness: ↑, CTX-1: ↑, Oc.N: ↔	[51]
Adenine-induced CKD mice	LiCl (150 mg/L drinking water, 6 weeks)	Calcium: ↔, phosphate: ↔, PTH: ↔, TRAP: ↔, BV/TV: ↑, Tb.N: ↔, Tb.Th: ↑, Tb.Sp: ↔	[24]
Ovariectomised rats	GSK3 inhibitor 603287-31-8 (3 mg/kg/day, oral, 2 months)	BV/TV: ↑, Tb.N: ↑, Tb.Th: ↑, Tb.Sp: ↓, cortical parameters: ↔, MS: ↔, MAR: ↑, BFR: ↔, load: ↑, work to failure: ↑, stiffness: ↑, Oc.N: ↔, OPG/RANKL: ↔	[8]
Rats with implant instability	GSK3 inhibitor AR28 (20 mg/kg/day, oral, 3 days)	BV/TV: ↑, Oc.N: ↓, TRAP: ↔, RANKL: ↔, OPG: ↑, RANKL/OPG: ↓	[52]
Titanium-stimulated calvariae osteolysis mice	LiCl (200 mg/kg/day, oral)	BMD: ↑, BV/TV: ↑, bone thickness: ↑, ES: ↓, TRAP-positive cells: ↓, Oc.S: ↓	[26]

Abbreviations: BFR: bone formation rate, BMC: bone mineral content, BMD: bone mineral density, BV/TV: bone volume/total volume, CKD: chronic kidney disease, Ct.Ar: cortical area, Ct.Th: cortical thickness, CTX-1: C-terminal crosslinking telopeptide of type I collagen, ES: eroded surface, GSK3β: glycogen synthase kinase-3 beta, LiCl: lithium chloride, MAR: mineral apposition rate, MS: mineralising surface, Oc.N: osteoclast number, Oc.S: osteoclast surface, OPG: osteoprotegerin, p-β-catenin: phosphorylated β-catenin, p-GSK3β: phosphorylated glycogen synthase kinase-3 beta, PTH: parathyroid hormone, RANKL: receptor activator of nuclear factor-kappa B ligand, Tb.N: trabecular number, Tb.Sp: trabecular separation, Tb.Th: trabecular thickness, TRAP: tartrate-resistant acid phosphatase, ↑: increase/upregulate, ↓: decrease/downregulate, ↔: no change.

**Table 5:** In vitro evidence for the effects of GSK3β inhibitor on osteoclastogenesis.

Type of Cells	Intervention and Concentration	Findings	Reference
Titanium-particle-stressed MC3T3-E1 cells	LiCl (10 mM)	OPG: ↑, RANKL: ↓, RANKL/OPG ratio: ↓, p-GSK3β: ↑	[26]
RAW-D cells	LiCl (10 mM) or SB216763 (10 μM)	TRAP-positive multinucleated cells: ↓, TRAP activity: ↓, NFATc1: ↓	[53]
M-CSF- and RANKL-stimulated BMMs	LY2090314 (GSK3β inhibitor) (3 μM)	TRAP-positive multinucleated cells: ↓, resorbed area: ↓, NFATc1: ↓, MMP9: ↓, CTSK: ↓, TRAP: ↓, CaN: ↓, TAK1: ↑, p-GSK3β: ↓	[46]
M-CSF- and RANKL-stimulated BMMs	LiCl (5 mM)	TRAP-positive multinucleated cells: ↑, p-GSK3β: ↑, NFATc1: ↑	[54]

Abbreviations: CaN: calcineurin, CTSK: cathepsin K, LiCl: lithium chloride, M-CSF: macrophage colony-stimulating factor, MC3T3-E1: murine pre-osteoblastic cells, MMP9: matrix metalloproteinase-9, NFATc1: nuclear factor of activated T-cells cytoplasmic 1, OPG: osteoprotegerin, p-GSK3β: phosphorylated glycogen synthase kinase-3 beta, RANKL: receptor activator of nuclear factor-kappa B ligand, RAW-D: murine osteoclast precursor, TAK1: transforming growth factor beta-activated kinase 1, TRAP: tartrate-resistant acid phosphatase, ↑: increase/upregulate, ↓: decrease/downregulate.

## 6. Perspectives

The regulation of osteoclastogenesis elicited through GSK3β represents a complex mechanism. The phosphorylation (inhibition) of GSK3β favours osteoclast formation, whereas the non-phosphorylation or dephosphorylation (activation) of GSK3β leads to suppression of osteoclastogenesis using osteoclast progenitors. The regulation of osteoclastogenesis by GSK3β was mediated via RANK/RANKL/OPG, PI3K/AKT, NF-κB, NRF2/KEAP1, canonical Wnt/β-catenin, and PKC signalling pathways. However, findings obtained from cell culture studies were not translated into an animal experimental model. Activation of GSK3β was associated with osteoporosis in animals induced by high-fat diet, glucocorticoid, and STZ. No effect on GSK3β level was observed in animals with oestrogen deficiency. The discrepancies between in vitro and in vivo studies could be attributed to the complexity of the skeletal microenvironment, consisting of bone formation and resorption processes governed by osteoblasts and osteoclasts, respectively. It was postulated that GSK3β activation in bone-forming cells and GSK3β inhibition in bone-resorbing cells occur concurrently. The reduction of GSK3β phosphorylation in osteoporotic conditions could be the net outcome of both actions.

Research gaps are identified based on the current state of knowledge. A wide array of research consistently indicated that GSK3β phosphorylation is required for osteoclast differentiation and activation. Despite numerous and consistent findings on the interaction between GSK3β and PI3K/AKT signalling molecules, studies investigating the crosstalk between GSK3β and other signalling molecules (NF-κB, NRF2/KEAP1, Wnt/β-catenin, and PKC) in orchestrating osteoclastogenesis are limited. The role of GSK3β in modulating NF-κB and NRF2/KEAP1 pathways is contradicted by existing knowledge. It remains uncertain whether GSK3β is a positive or negative regulator for these signalling networks during osteoclastogenesis. Furthermore, the levels of pro- and anti-inflammatory mediators are not measured during osteoclastogenesis in inflammatory conditions. The overexpression and silencing of GSK3β in cell culture and animal experimental models should represent the next step of research to address the direct action of GSK3β on osteoclast formation and bone resorption. The current review offers a notably strong and comprehensive overview of the role of GSK3β in osteoclast formation and bone resorption. However, the lack of discussion on osteogenesis and bone formation represents a limitation of this review.

## 7. Conclusions

The phosphorylation (inhibition) of GSK3β favours the differentiation of osteoclast precursors into osteoclasts and their multinucleation into mature osteoclasts in the presence of M-CSF and RANKL. Nonetheless, GSK3β inhibition is associated with better bone health in animals, which could be the net outcome for the phosphorylation status of GSK3β in osteoblasts and osteoclasts. These observations also suggest that the actions of GSK3β may be more prominent in bone formation than bone resorption.
